# One-year weight loss and metabolic outcomes after laparoscopic sleeve gastrectomy in adolescents compared with young adults: A propensity score-matched cohort study

**DOI:** 10.1016/j.obpill.2026.100315

**Published:** 2026-08-07

**Authors:** Ahmed Taki-Eldin, Ahmed Lotfy, Mohamed saif Eldin Mahmoud El-Soudani, Mahmoud Abuelyazeed Ghaly, Mohamed Mahmoud Mohamed Eltaweel, Eslam Taha Ghalwash, Tamer Rushdy Elalfy, Ahmed M. Kandel, Zakaria M. Abdel Sadek, Ahmed Naji Ahmed

**Affiliations:** a General Surgery Department, Faculty of Medicine, Horus University, Egypt, New Damietta, Egypt; b General Surgery Department, Faculty of Medicine, Mansoura University, Mansoura, Dakahlia, Egypt; c Department of Pediatric Surgery, Al-Azhar University, Faculty of Medicine, Cairo, Egypt; d General Surgery Department, Faculty of Medicine, Azhar University, Cairo, Egypt; e General Surgery Department, Faculty of Medicine for Girls, Al-Azhar University, New Damietta, Egypt; f General Surgery Department, Faculty of Medicine, Zagazig University, Zagazig, Egypt; g General Surgery Department, Faculty of Medicine, Al-Azhar University, New Damietta, Egypt; h Pediatric Department, Faculty of Medicine for Girls, Al-Azhar University, Assiut, Egypt; i Faculty of Medicine Sudan University of Science and Technology, Khartoum, Sudan

**Keywords:** Sleeve gastrectomy, Adolescent obesity, Bariatric surgery, Propensity score matching, GLP-1 receptor agonist, TriNetX, Severe obesity, Metabolic outcomes

## Abstract

**Introduction:**

Adolescent bariatric surgery is rising, but comparative outcome data for sleeve gastrectomy in adolescents and young adults remain limited. Pre-surgical glucagon-like peptide-1 receptor agonist use in younger patients represents an unaddressed confounder. We therefor compared 1-year weight loss, metabolic comorbidity, nutritional and surgical outcomes of laparoscopic SG between adolescents (13–17 years) and young adults (18–21years) with severe obesity, using propensity score matching (PSM).

**Methods:**

This retrospective comparative cohort study used the TriNetX US Collaborative Network,a fedrated electronic health record platform spanning 66 health care organizations and approximately 113 million patients. Patients who underwent laparoscopic SG with morbid obesity were identified and stratified by age group. After exclusion of prior or concurrent alternative bariatric procedures, adolescents (n = 327) and young adults (n = 1056) were matched 1:1 using 25 pre-surgical characteristics, including GLP-1 RA medications. Outcomes were assessed at 1 year.

**Results:**

After PSM, 318 matched pairs were analyzed. Pre-surgical semaglutide use was markedly higher among adolescents (29.4% vs 14.6%; SMD 0.363 before matching) and was balanced after matching (27.4% vs 26.1%; SMD 0.028). At 1-year, post-operative Body Mass Index (BMI) was equivalent (38.6 ± 8.7 vs 39.1 ± 8.8 kg/m^2^; p = 0.534). No significant differences were observed in type 2 diabetes persistence, hypertension persistence, dyslipidemia, NAFLD, or new-onset GERD. No reoperations or conversions to RYGB occurred at 1 year. Approximately 49% of patients in both cohorts had pre-existing vitamin D insufficiency. Sex-stratified analyses were concordant with primary findings.

**Conclusions:**

Adolescents achieve equivalent 1-year weight loss and metabolic outcomes following SG compared with young adults. Pre-surgical GLP-1 RA exposure is substantially higher in adolescents and must be controlled for in comparative bariatric research. The high prevalence of pre-existing vitamin D insufficiency in this population warrants routine pre-operative assessment and supplementation.

## Graphical abstract

**Figure:**
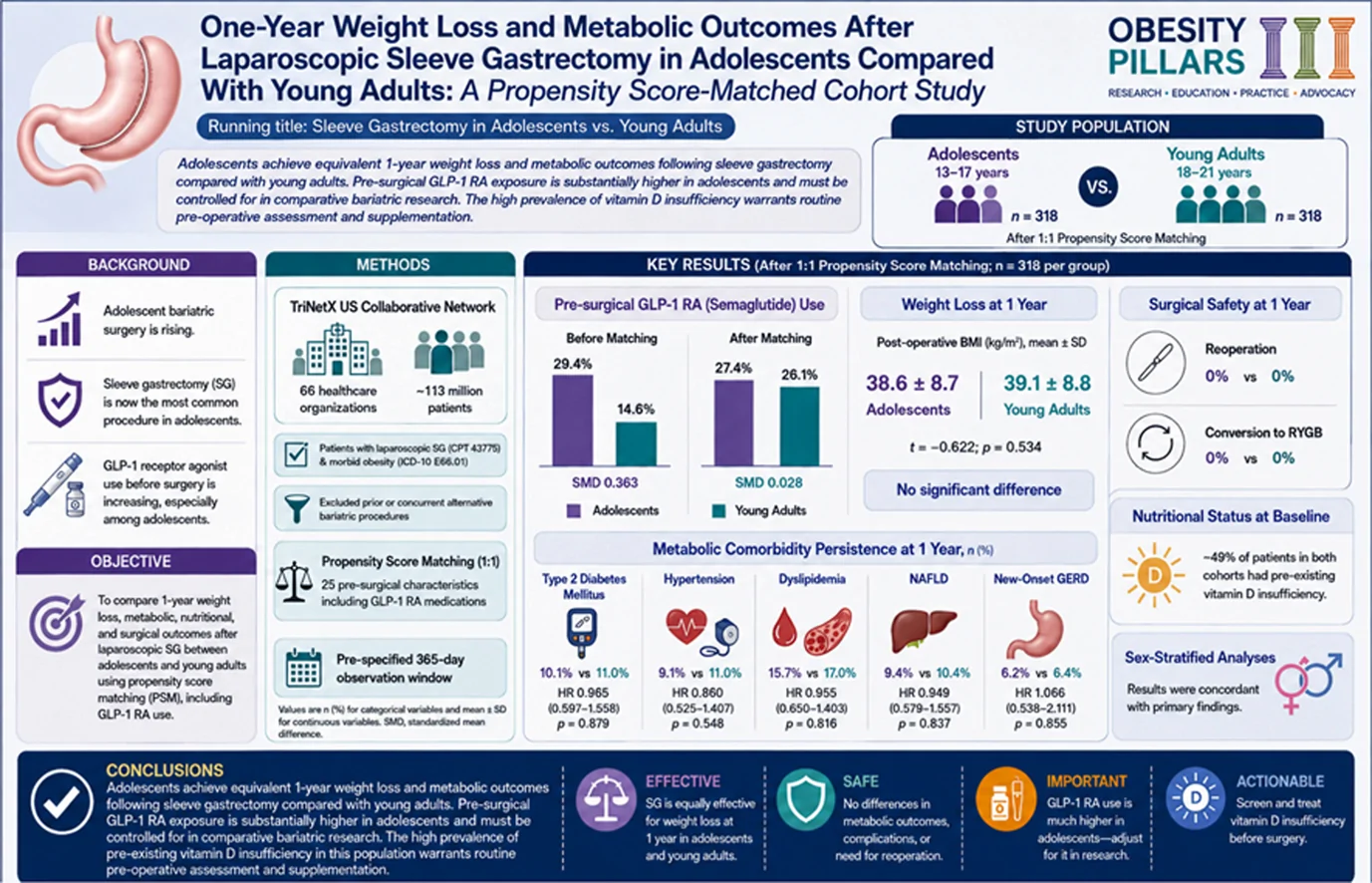

## 1 Introduction

Severe obesity in children and adolescents has reached a critical public health threshold in the United States, with prevalence estimates exceeding 9% of the pediatric population and increasing trajectories of associated cardiometabolic disease [1]. For adolescents in whom lifestyle modification and pharmacotherapy have failed, bariatric surgery remains the most effective and durable intervention, offering measurable improvements in weight, metabolic health, and quality of life [2,3]. Over the past decade, sleeve gastrectomy (SG) has emerged as the dominant bariatric procedure in adolescents, supplanting Roux-en-Y gastric bypass (RYGB) in US registries due to its technical simplicity, lack of intestinal rerouting, and reversibility [3,4] Prospective adolescent series, including the Swedish AMOS study and the long-term Teen-LABS follow-up, have established durable weight loss and comorbidity benefit out to 5 years following bariatric surgery [5,6].

Despite growing surgical volumes, comparative outcome data stratified by age at the time of surgery remain limited. Adolescents represent a physiologically distinct group: they are in active somatic growth, possess greater pancreatic beta-cell plasticity, and carry higher micronutrient demands relative to adults [7,8]Young adults aged 18–21 years, while legally autonomous, share many of these physiological characteristics yet are routinely grouped with older adult bariatric populations in published literature. The severity of obesity-related cardiometabolic risk in this young population further heightens the clinical stakes of surgical decision-making [9] Whether age at SG within this 13–21 year transition window independently influences short-term outcomes has not been systematically addressed in a propensity-adjusted, nationally representative cohort.

A critical and underappreciated methodological issue in contemporary adolescent bariatric research is the unprecedented adoption of glucagon-like peptide-1 receptor agonists (GLP-1 RAs), particularly semaglutide, in the adolescent obesity population. Following FDA approval of once-weekly semaglutide (Wegovy®) for patients aged 12 years and older in December 2022 [10],pre-surgical GLP-1 RA use among adolescent bariatric candidates has increased substantially. Since GLP-1 RAs independently reduce BMI, improve glycemic control, and alter hepatic steatosis [11,12] their pre-surgical use constitutes a direct confounder of post-operative metabolic outcomes when not controlled for in analytical models. To our knowledge, no prior database-based bariatric study has explicitly incorporated pre-surgical GLP-1 RA exposure as a propensity score matching variable.

We conducted a propensity score-matched retrospective cohort study using the TriNetX US Collaborative Research Network to compare 1-year outcomes of laparoscopic SG in adolescents (aged 13–17 years) versus young adults (aged 18–21 years) with severe obesity, with dedicated adjustment for pre-surgical GLP-1 RA use and other clinically relevant confounders.

## 2 Methods

### 2.1 Study design and data source

This was a retrospective, propensity score-matched, comparative cohort study of de-identified electronic health record data. We used the TriNetX US Collaborative Research Network, a federated electronic health record (EHR) analytics platform aggregating longitudinal de-identified data from 66 US healthcare organizations, representing approximately 113 million patients across academic medical centers, community hospitals, and integrated health systems. The platform enables cohort identification, propensity score matching, and outcomes analysis while maintaining data privacy through aggregation thresholds (minimum cell size of 11 patients per reported stratum). Institutional review board approval was not required, as TriNetX provides access to de-identified, aggregate-level data classified as exempt from human subjects research under 45 CFR 46.101(b) [4].

As a secondary analysis of de-identified, pre-existing electronic health record data this study did not involve direct patient contact, intervention, or collection of new biospecimens. Consequently individual patient consent was neither required nor obtainable as all data were fully de-identified prior to platform access. For minors included in the database, the original treating institutions obtained appropriate informed consent from patients and/or their legally authorized representatives at the time of clinical care and data collection. This manuscript was prepared in accordance with the Strengthening the Reporting of Observational Studies in Epidemiology (STROBE) checklist [13].

### 2.2 Cohort identification

The study population comprised patients who underwent laparoscopic sleeve gastrectomy, identified by CPT procedure code 43775 (Laparoscopy, surgical, gastric restrictive procedure; longitudinal gastrectomy), in the setting of morbid obesity (ICD-10-CM E66.01: Morbid [severe] obesity due to excess calories). To ensure procedure specificity, we applied comprehensive exclusion criteria in the CANNOT HAVE domain, excluding all patients with any prior or concurrent alternative bariatric procedure: Roux-en-Y gastric bypass (CPT 43644, 43645, 43846, 43847), laparoscopic adjustable gastric banding (CPT 43770), biliopancreatic diversion with duodenal switch (CPT 43845), vertical banded gastroplasty (CPT 43842), and revision bariatric surgery (CPT 43848). The index event was defined as the first date on which all inclusion criteria were simultaneously satisfied.

Two cohorts were defined by patient age at the index event: Cohort 1, *SG Adolescents* (aged 13–17 years), and Cohort 2, *SG Young Adults* (aged 18–21 years). The upper age boundary of 21 years was selected to capture the transition-age population that shares clinical and physiological characteristics with adolescents while being managed within adult bariatric programs. The date range of the query encompassed available TriNetX data through June 2026, capturing the contemporary era of adolescent bariatric surgery including the post-semaglutide approval period.

### 2.3 Propensity score matching

To minimize confounding by indication and account for systematic differences between age groups, we implemented 1:1 nearest-neighbor propensity score matching (PSM) with a caliper of 0.2 standard deviations of the logit of the propensity score within the TriNetX analytics platform [14,15]The propensity score was estimated from 25 pre-surgical patient characteristics assessed in a 365-day baseline window prior to the index surgical date.

Matching variables were selected on clinical grounds and included: (i) seven baseline diagnoses — sleep disorders (G47), dyslipidemia (E78.5), depressive episode (F32), type 2 diabetes mellitus (E11), essential hypertension (I10), non-alcoholic fatty liver disease (K76.0), and gastro-esophageal reflux disease (K21); (ii) fourteen continuous laboratory values — BMI, body weight, systolic and diastolic blood pressure, HbA1c, total cholesterol, LDL cholesterol, HDL cholesterol, triglycerides, serum albumin, calcidiol (vitamin D), ferritin, vitamin B12, and serum iron; and (iii) four pre-surgical medication exposures — semaglutide, metformin, liraglutide, and insulin. The deliberate inclusion of semaglutide as a PSM covariate was a pre-planned methodological decision, prompted by the observed 2-fold higher prevalence of pre-surgical semaglutide use in adolescents (29.4% vs 14.6%) — an imbalance that, if uncorrected, would bias post-operative weight and metabolic outcome comparisons. Balance was assessed using standardized mean differences (SMD), with SMD <0.10 defined as adequate balance.

### 2.4 Outcome measures

All outcomes were assessed within a pre-specified post-operative window of 1–365 days following the index surgical date.

The **primary outcome** was post-operative BMI at 1-year follow-up, captured as the most recent BMI measurement recorded within the observation window.

Secondary outcomes included: (i) **body weight** at 1 year; (ii) **metabolic comorbidity persistence** — defined as the presence of a diagnosis code for type 2 diabetes mellitus, hypertension, dyslipidemia, or NAFLD at any time during the follow-up window (without excluding patients with pre-operative diagnosis, to capture persistent disease rather than incident disease); (iii) **new-onset complications** — including GERD (K21), vitamin B12 deficiency (E53.8), vitamin D deficiency (E55.9), iron deficiency anemia (D50.9), and protein-calorie malnutrition (E46), each analyzed exclusively in patients without the respective pre-operative diagnosis (prior outcomes excluded); and (iv) **surgical safety outcomes** — reoperation (CPT 43848) and conversion to RYGB (CPT 43644).

### 2.5 Sex-stratified subgroup analysis

A pre-specified sex-stratified analysis was conducted by constructing four separate cohorts: adolescent females (n = 226), young adult females (n = 735), adolescent males (n = 101), and young adult males (n = 321). Within each sex stratum, independent PSM was performed using the same matching framework described above, with the age variable excluded from the model to permit matching between age groups. The male subgroup analysis (n = 95 matched pairs) is designated as exploratory given residual imbalance in BMI (SMD 0.273) and smaller sample size.

### 2.6 Statistical analysis

All statistical analyses were performed within the TriNetX analytics platform (version accessed June 2026). Continuous outcomes (BMI, body weight) were compared using independent-samples t-tests. Binary outcomes were assessed using risk ratios (RR) and odds ratios (OR) with 95% confidence intervals and risk differences (RD). Time-to-event analyses were performed using Kaplan-Meier survival estimation with log-rank tests and Cox proportional hazards regression, generating hazard ratios (HR) with 95% confidence intervals. Proportionality of hazards was assessed using the Schoenfeld residuals test. A 365-day pre-specified observation window was applied to minimize differential follow-up bias identified during preliminary analysis (median follow-up: adolescents 301 days vs young adults 365 days at 1-year truncation). A two-sided p-value <0.05 was considered statistically significant for all primary comparisons. Data are expressed as mean ± standard deviation or n (%) unless otherwise stated. Outcomes with fewer than 11 events per group could not be reported due to TriNetX platform privacy thresholds.

## 3 Results

### 3.1 Study population and baseline characteristics

A total of 327 adolescents (aged 13–17 years) and 1056 young adults (aged 18–21 years) with severe obesity who underwent laparoscopic SG were identified across the TriNetX US Collaborative Network. After 1:1 propensity score matching, 318 matched pairs were retained (97.2% of the smaller cohort), yielding a final analytic sample of 636 patients.

Baseline characteristics before and after matching are presented in Table 1. Before matching, young adults had higher prevalence rates of NAFLD (18.6% vs 11.6%; p = 0.003), GERD (16.0% vs 10.4%; p = 0.012), and hypertension (16.3% vs 11.9%; p = 0.055) differences plausibly attributable to the longer cumulative duration of obesity exposure. Conversely, adolescents exhibited nearly double the rate of pre-surgical semaglutide use (29.4% vs 14.6%; p < 0.001; SMD 0.363), reflecting the 2022 FDA approval of semaglutide for patients aged 12 years and older.

**Table 1:** Baseline patient characteristics before and after 1:1 propensity score matching.

Characteristic	Before Matching			After 1:1 Propensity Score Matching			
	Adolescents (n = 327)	Young Adults (n = 1056)	SMD	Adolescents (n = 318)	Young Adults (n = 318)	SMD	p-value
**Demographics**							
**Female sex, n (%)**	226 (69.1%)	735 (69.6%)	–	218 (68.6%)	218 (68.6%)	<0.001	–
**Age at index surgery, years**	14.3 ± 1.6	16.9 ± 1.6	1.621	–	–	–	–
**Baseline Diagnoses, n (%)**							
**Sleep disorders (G47)**	180 (55.0%)	492 (46.6%)	0.170	172 (54.1%)	161 (50.6%)	0.069	0.382
**Dyslipidemia (E78.5)**	80 (24.5%)	223 (21.1%)	0.080	75 (23.6%)	77 (24.2%)	0.015	0.852
**Depressive episode (F32)**	62 (19.0%)	200 (18.9%)	<0.001	60 (18.9%)	51 (16.0%)	0.075	0.347
**Type 2 diabetes mellitus (E11)**	39 (11.9%)	152 (14.4%)	0.073	39 (12.3%)	35 (11.0%)	0.039	0.621
**Hypertension (I10)**	39 (11.9%)	172 (16.3%)	0.126	39 (12.3%)	30 (9.4%)	0.091	0.251
**NAFLD (K76.0)**	38 (11.6%)	196 (18.6%)	0.195	38 (12.0%)	32 (10.1%)	0.060	0.447
**GERD (K21)**	34 (10.4%)	169 (16.0%)	0.166	34 (10.7%)	36 (11.3%)	0.020	0.800
**Baseline Laboratory Values, Mean ± SD**							
**BMI (kg/m^2^)**	47.2 ± 7.2	48.1 ± 7.4	0.114	47.3 ± 7.2	48.4 ± 7.3	0.160	0.057
**Body weight (lbs)**	289 ± 57	295 ± 61	0.109	289 ± 58	297 ± 58	0.130	0.129
**Systolic BP (mmHg)**	123 ± 14	124 ± 13	0.056	123 ± 14	122 ± 13	0.061	0.460
**Diastolic BP (mmHg)**	72 ± 10	74 ± 11	0.146	72 ± 10	72 ± 11	0.016	0.844
**HbA1c (%)**	5.59 ± 0.93	5.58 ± 0.91	0.009	5.59 ± 0.94	5.52 ± 1.04	0.074	0.471
**Total cholesterol (mg/dL)**	161 ± 31	164 ± 34	0.103	161 ± 30	163 ± 36	0.047	0.656
**LDL cholesterol (mg/dL)**	95 ± 26	100 ± 29	0.184	95 ± 26	99 ± 32	0.132	0.220
**HDL cholesterol (mg/dL)**	42 ± 10	42 ± 10	0.029	42 ± 10	43 ± 10	0.023	0.826
**Triglycerides (mg/dL)**	125 ± 74	122 ± 82	0.044	125 ± 75	110 ± 55	0.237	0.028
**Albumin (g/dL)**	4.19 ± 0.45	4.21 ± 0.42	0.055	4.19 ± 0.45	4.20 ± 0.43	0.021	0.833
**Vitamin D/calcidiol (ng/mL)**	21.1 ± 9.5	20.9 ± 10.9	0.011	21.0 ± 9.5	18.3 ± 8.1	0.309	0.081
**Ferritin (ng/mL)**	43.5 ± 34	64.2 ± 80	0.336	43.8 ± 34	71.3 ± 102	0.363^a^	0.007
**Vitamin B12 (pg/mL)**	418 ± 144	464 ± 222	0.245	418 ± 144	452 ± 192	0.202	0.143
**Serum iron (μg/dL)**	60 ± 26	61 ± 29	0.014	60 ± 26	60 ± 27	0.014	0.919
**Pre-surgical Medications, n (%)**							
**Semaglutide (RxNorm 1991302)**	96 (29.4%)	154 (14.6%)	0.363	87 (27.4%)	83 (26.1%)	0.028	0.720
**Metformin**	54 (16.5%)	206 (19.5%)	0.078	54 (17.0%)	49 (15.4%)	0.043	0.590
**Liraglutide**	20 (6.1%)	63 (6.0%)	0.006	20 (6.3%)	13 (4.1%)	0.099	0.211
**Insulin**	10 (3.1%)	31 (2.9%)	0.007	10 (3.1%)	10 (3.1%)	<0.001	1.000
**Follow-up Duration After Matching**							
**Median follow-up (days)**	–	–	–	301	365	–	–
**Mean follow-up ± SD (days)**	–	–	–	247 ± 132	287 ± 117	–	–

Values are n (%) for categorical variables and Mean ± SD for continuous variables. SMD, standardized mean difference; BMI, body mass index; BP, blood pressure; NAFLD, non-alcoholic fatty liver disease; GERD, gastro-esophageal reflux disease; GLP-1 RA, glucagon-like peptide-1 receptor agonist.

a Ferritin was not a primary propensity score matching variable; residual imbalance after matching is noted as a study limitation.

**Fig. 1:**
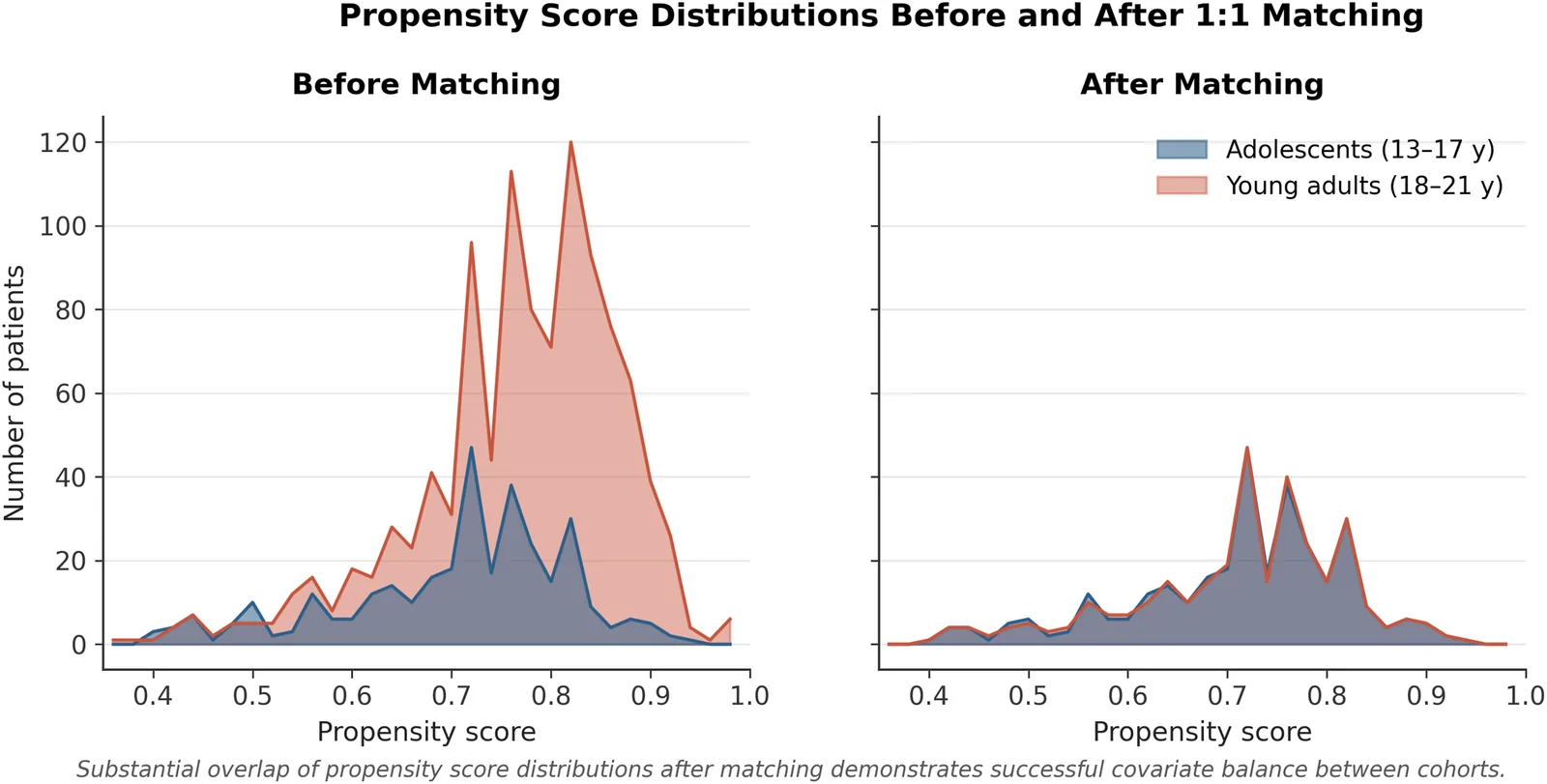
Propensity score distributions before and after 1:1 matching. Histograms display the number of patients across propensity score values for adolescents (blue) and young adults (orange). Before matching (left), young adults predominate at higher propensity scores; after matching (right), the distributions overlap substantially, demonstrating successful covariate balance. (For interpretation of the references to colour in this figure legend, the reader is referred to the Web version of this article.)

After propensity score matching, all seven diagnostic variables and all four medication exposures had SMDs <0.10, including semaglutide (27.4% vs 26.1%; SMD 0.028). The substantial overlap of propensity score distributions after matching (Fig. 1) confirmed adequate covariate balance. Most laboratory values were similarly balanced, except for ferritin (43.8 ± 34 vs 71.3 ± 102 ng/mL; SMD 0.363) and triglycerides (125 ± 75 vs 110 ± 55 mg/dL; SMD 0.237), which were not primary PSM covariates. Residual BMI imbalance was modest (47.3 ± 7.2 vs 48.4 ± 7.3 kg/m^2^; SMD 0.160). A clinically significant finding emerging from the baseline evaluation was the high prevalence of vitamin D insufficiency in both cohorts: mean calcidiol levels were below the sufficient threshold in both groups (21.0 ± 9.5 vs 18.3 ± 8.1 ng/mL), and approximately 49% of patients in each cohort met criteria for pre-existing vitamin D deficiency at the time of surgery. After matching, median follow-up was 301 days (mean 247 ± 132) for adolescents and 365 days (mean 287 ± 117) for young adults within the 365-day window.

### 3.2 Primary outcome: weight loss at 1 year

**Fig. 2:**
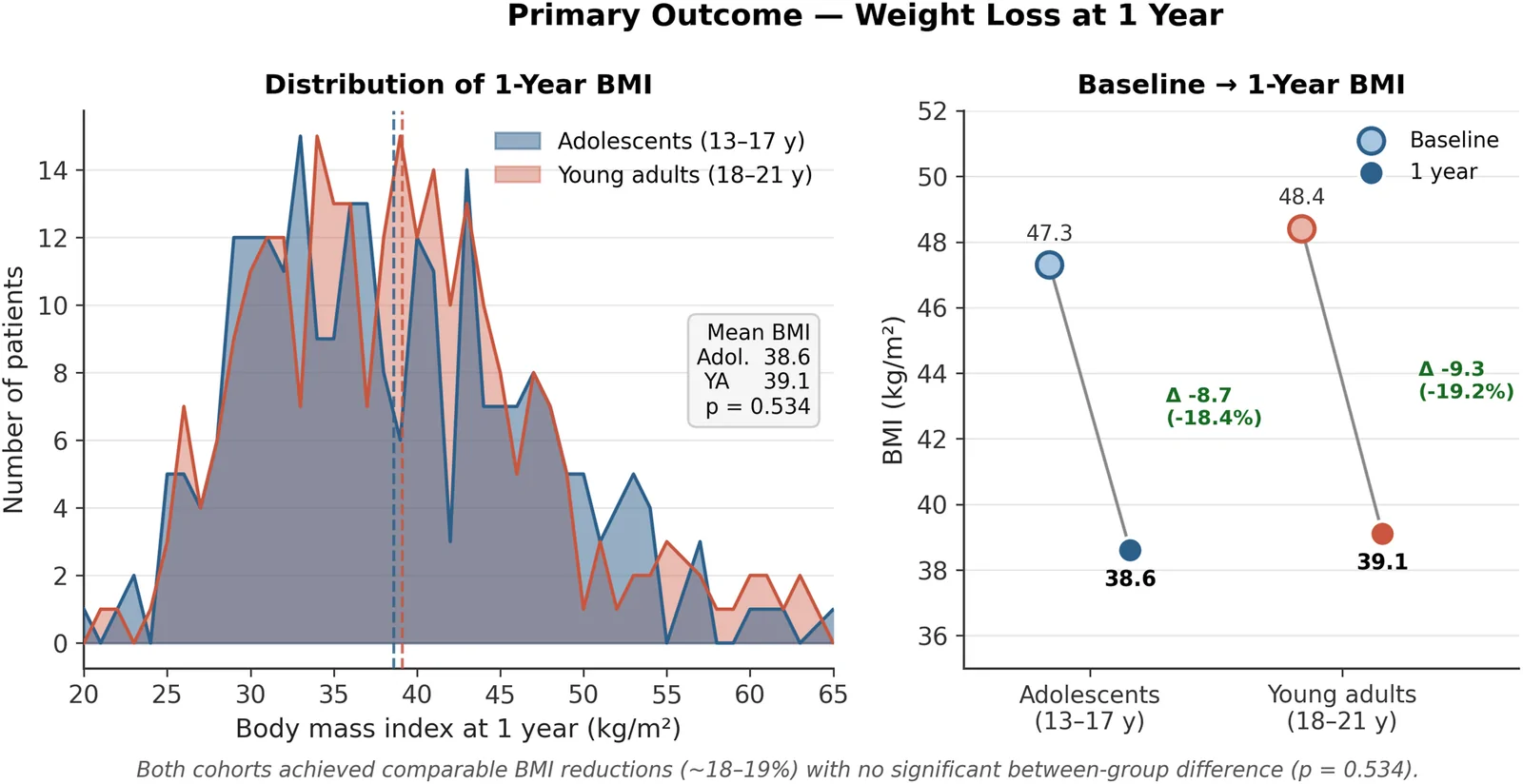
Primary outcome — weight loss at 1 year. Left: distribution of 1-year body mass index (BMI) for both cohorts, with vertical dashed lines indicating cohort means (38.6 vs 39.1 kg/m^2^; p = 0.534). Right: baseline-to-1-year BMI trajectory, showing comparable absolute and percentage reductions in both groups (−18.4% vs −19.2%).

Post-operative BMI at 1-year follow-up did not differ significantly between adolescents and young adults (38.6 ± 8.7 vs 39.1 ± 8.8 kg/m^2^; t = −0.622; df = 519; p = 0.534; Table 2, Fig. 2). Body weight was likewise equivalent (237 ± 63 vs 240 ± 61 lbs; t = −0.505; p = 0.613). Both cohorts achieved substantial BMI reductions from their pre-operative baseline: adolescents from 47.3 to 38.6 kg/m^2^ (ΔBMI −8.7 kg/m^2^; −18.4%) and young adults from 48.4 to 39.1 kg/m^2^ (ΔBMI −9.3 kg/m^2^; −19.2%). Corresponding bariatric weight loss metrics were 39.0% excess BMI loss (EBMIL) and 18.0% total body weight loss (TBWL) in adolescents, and 39.7% EBMIL and 19.2% TBWL in young adults. These reductions were comparable in magnitude and not statistically different between groups.

**Table 2:** Post-operative Outcomes at 1-Year Follow-up: Propensity Score-Matched Main Analysis (n = 318 per group).

Outcome	Adolescents (n = 318)	Young Adults (n = 318)	Risk Ratio or t-statistic (95% CI)	Hazard Ratio (95% CI)	p-value
**Primary Outcome: Weight Loss at 1 Year, Mean ± SD**					
BMI (kg/m^2^)	38.6 ± 8.7	39.1 ± 8.8	t = −0.622	–	**0.534**
Body weight (lbs)	237 ± 63	240 ± 61	t = −0.505	–	**0.613**
**Metabolic Comorbidity Persistence, n (%)**^a^					
Type 2 diabetes mellitus	32 (10.1%)	35 (11.0%)	RR 0.91 (0.58–1.44)	HR 0.965 (0.597–1.558)	**0.879**
Hypertension	29 (9.1%)	35 (11.0%)	RR 0.83 (0.52–1.32)	HR 0.860 (0.525–1.407)	**0.548**
Dyslipidemia	50 (15.7%)	54 (17.0%)	RR 0.93 (0.65–1.32)	HR 0.955 (0.650–1.403)	**0.816**
NAFLD	30 (9.4%)	33 (10.4%)	RR 0.91 (0.57–1.45)	HR 0.949 (0.579–1.557)	**0.837**
**New-Onset Complications (Excluding Patients with Prior Diagnosis), n (%)**^b^					
GERD (new onset)	16/259 (6.2%)	17/264 (6.4%)	RR 0.96 (0.50–1.86)	HR 1.066 (0.538–2.111)	**0.855**
Vitamin B12 deficiency	12/315 (3.8%)	15/309 (4.9%)	RR 0.78 (0.37–1.65)	HR 0.936 (0.438–2.001)	**0.865**
Vitamin D deficiency	–	–	–	–	**Insufficient events (<11)**
Iron deficiency anemia	–	–	–	–	**Insufficient events (<11)**
Protein-calorie malnutrition	–	–	–	–	**Insufficient events (<11)**
**Surgical Safety Outcomes at 1 Year**					
Reoperation (CPT 43848)	0/318 (0%)	0/318 (0%)	–	–	**—**
Conversion to RYGB (CPT 43644)	0/318 (0%)	0/318 (0%)	–	–	**—**

RR, risk ratio; HR, hazard ratio; CI, confidence interval; RYGB, Roux-en-Y gastric bypass; NAFLD, non-alcoholic fatty liver disease; GERD, gastro-esophageal reflux disease.

a Metabolic comorbidity persistence outcomes were assessed without excluding patients with pre-operative diagnosis (to reflect disease persistence rather than incidence).

b New-onset complication outcomes excluded patients with the respective pre-operative diagnosis. Outcomes with insufficient events (<11 per group per TriNetX privacy threshold) are reported as not calculable.

### 3.3 Metabolic comorbidity outcomes

**Fig. 3:**
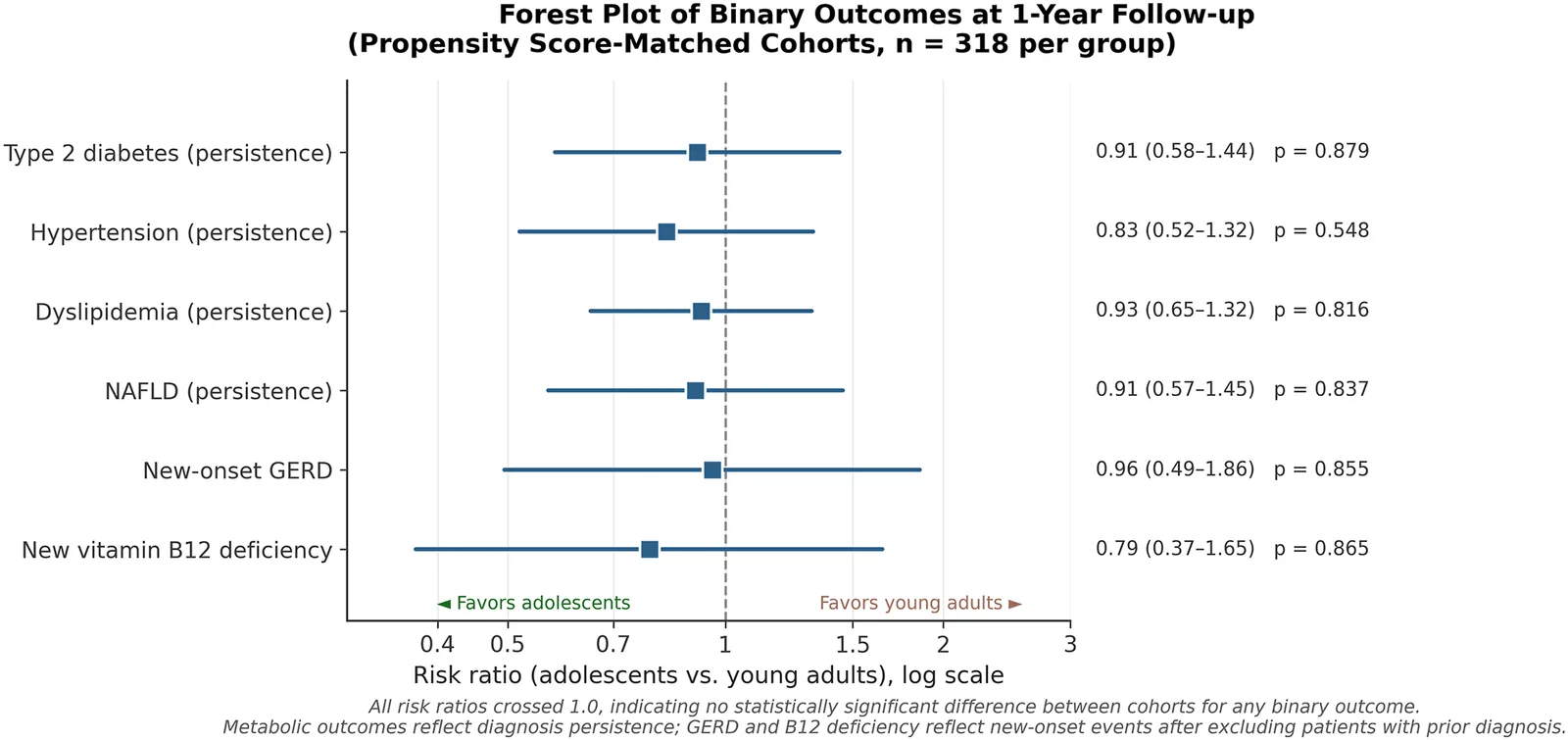
Forest plot of binary outcomes at 1-year follow-up. Risk ratios (squares) with 95% confidence intervals (horizontal lines) for adolescents versus young adults, plotted on a logarithmic scale. The vertical dashed line indicates a risk ratio of 1.0 (no difference). All confidence intervals cross 1.0, indicating no statistically significant between-group difference for any binary outcome.

**Fig. 4:**
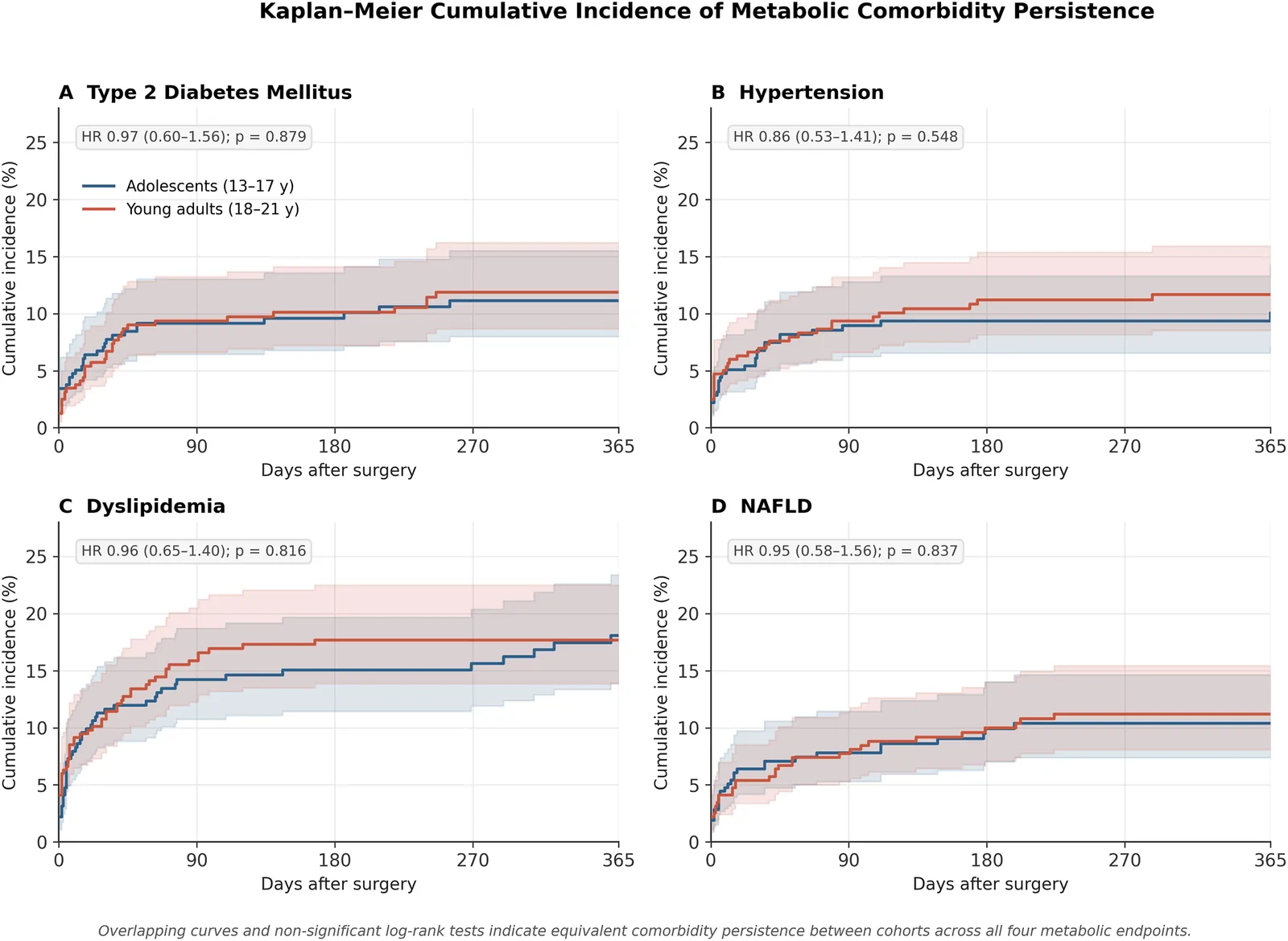
Kaplan–Meier cumulative incidence of metabolic comorbidity persistence. Cumulative incidence curves over 365 days for (A) type 2 diabetes mellitus, (B) hypertension, (C) dyslipidemia, and (D) non-alcoholic fatty liver disease. Shaded bands represent 95% confidence intervals. Hazard ratios and log-rank p-values are inset within each panel. Overlapping curves indicate equivalent comorbidity persistence between cohorts.

No statistically significant difference was observed in the persistence of any metabolic comorbidity between adolescents and young adults at 1 year (Fig. 3). Type 2 diabetes mellitus persisted in 10.1% of adolescents versus 11.0% of young adults (RR 0.91, 95% CI 0.58–1.44; HR 0.965, 95% CI 0.597–1.558; log-rank p = 0.879). Hypertension persisted in 9.1% versus 11.0% (HR 0.860, 95% CI 0.525–1.407; p = 0.548). Dyslipidemia rates were 15.7% versus 17.0% (HR 0.955, 95% CI 0.650–1.403; p = 0.816), and NAFLD rates were 9.4% versus 10.4% (HR 0.949, 95% CI 0.579–1.557; p = 0.837). Kaplan-Meier cumulative incidence curves were closely overlapping for all four metabolic endpoints, with no significant log-rank differences (Fig. 4).

### 3.4 New-onset complications

Among patients without pre-operative GERD (n = 259 adolescents; n = 264 young adults after excluding 59 and 54 patients with prior diagnosis, respectively), new-onset GERD developed in 6.2% of adolescents and 6.4% of young adults within 1 year (HR 1.066, 95% CI 0.538–2.111; log-rank p = 0.855). These rates are consistent with published early GERD incidence data following SG(16).

New vitamin B12 deficiency, after excluding 10 patients per group with pre-existing deficiency, occurred in 3.8% of adolescents and 4.9% of young adults (HR 0.936, 95% CI 0.438–2.001; log-rank p = 0.865). Vitamin D deficiency, iron deficiency anemia, and protein-calorie malnutrition yielded fewer than 11 events per group within the 1-year window, precluding formal statistical comparison. This finding is consistent with the known delayed presentation of nutritional complications after SG, which typically manifest at 2–4 years post-operatively and require longer follow-up for adequate characterization [16].

### 3.5 Surgical safety outcomes

No reoperations (CPT 43848) and no conversions from SG to RYGB (CPT 43644) were recorded in either cohort within 365 days of the index procedure. This finding, while limited by the relatively short observation window and the possibility of unrecorded out-of-network procedures in the EHR, suggests comparable early surgical safety across age groups.

### 3.6 Sex-stratified subgroup analysis

**Fig. 5:**
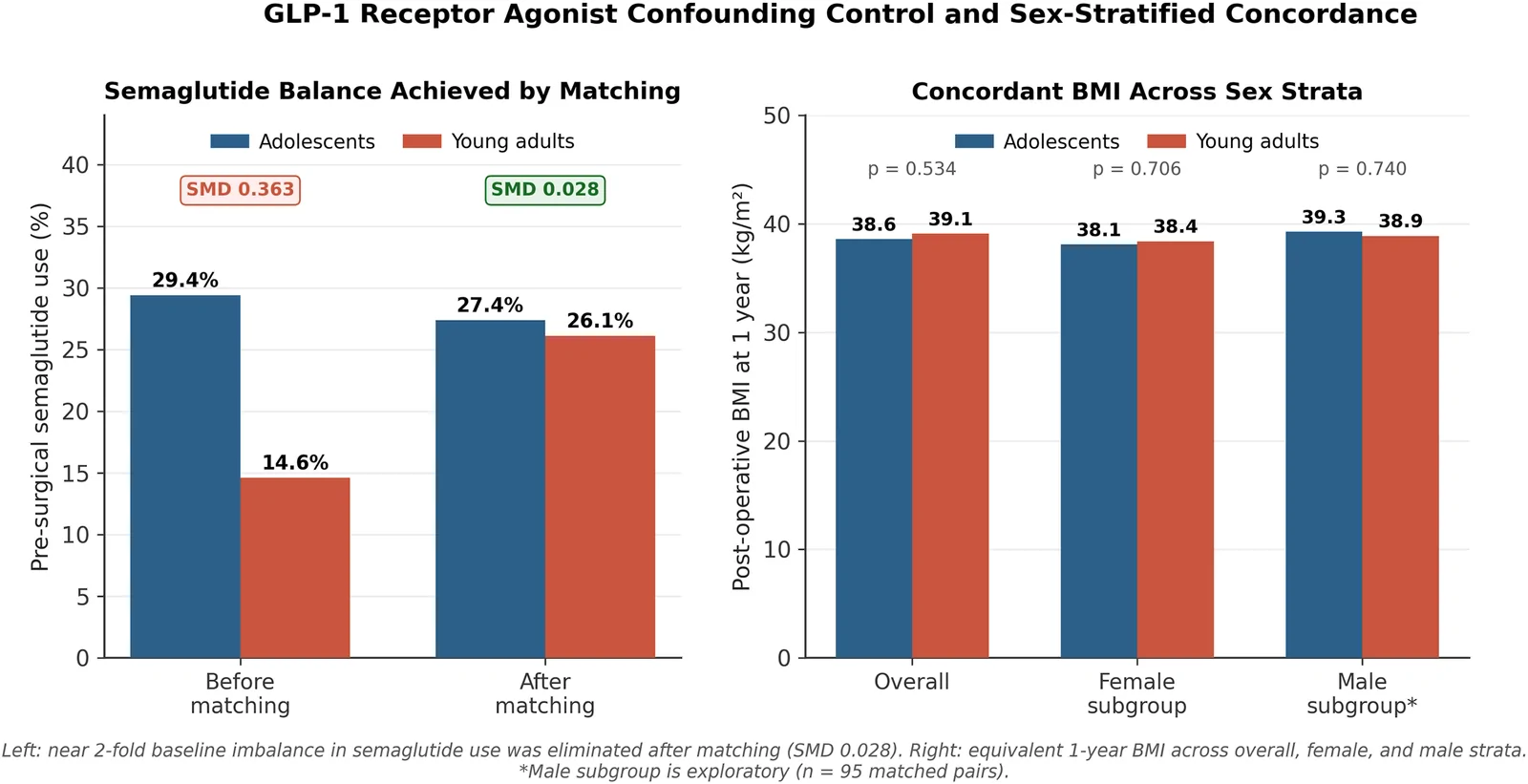
GLP-1 receptor agonist confounding control and sex-stratified concordance. Left: pre-surgical semaglutide use before and after matching, demonstrating elimination of the near 2-fold baseline imbalance (standardized mean difference reduced from 0.363 to 0.028). Right: post-operative 1-year BMI across the overall cohort and within female and male sex strata, showing concordant equivalence in all three comparisons. The male subgroup is exploratory (n = 95 matched pairs).

Table 3 presents outcomes stratified by sex. Among 218 matched female pairs, post-operative BMI was 38.1 ± 8.2 versus 38.4 ± 8.3 kg/m^2^ (p = 0.706), and T2D persisted in 11.0% of adolescent females versus 9.2% of young adult females (p = 0.525). NAFLD was slightly lower in adolescent females (7.3% vs 9.6%; p = 0.390). Among 95 matched male pairs (exploratory), post-operative BMI was 39.3 ± 9.1 versus 38.9 ± 7.2 kg/m^2^ (p = 0.740), and hypertension persisted in 18.9% versus 22.1% (p = 0.590). No outcome in either sex-stratified analysis reached statistical significance, and all findings were directionally consistent with the primary analysis (Fig. 5).

**Table 3:** Sex-stratified subgroup analysis of 1-year post-operative outcomes.

Outcome	Female Subgroup (n = 218 matched pairs)			Male Subgroup* (n = 95 matched pairs)		
	Adolescent F (n = 218)	Young Adult F (n = 218)	p-value	Adolescent M (n = 95)	Young Adult M (n = 95)	p-value
**Weight Outcomes, Mean ± SD**						
BMI (kg/m^2^)	38.1 ± 8.2	38.4 ± 8.3	**0.706**	39.3 ± 9.1	38.9 ± 7.2	**0.740**
Body weight (lbs)	225 ± 54	231 ± 60	**0.370**	262 ± 70	268 ± 53	**0.603**
**Metabolic Comorbidity Persistence, n (%)**						
Type 2 diabetes mellitus	24 (11.0%)	20 (9.2%)	**0.525**	<11 events^a^	–	**—**
Hypertension	<11 events^a^	–	**—**	18 (18.9%)	21 (22.1%)	**0.590**
Dyslipidemia	34 (15.6%)	38 (17.4%)	**0.606**	14 (14.7%)	12 (12.6%)	**0.673**
NAFLD	16 (7.3%)	21 (9.6%)	**0.390**	13 (13.7%)	12 (12.6%)	**0.830**
New-onset GERD	<11 events^a^	–	**—**	<11 events^a^	–	**—**

Female subgroup: 218 propensity score-matched pairs per group. Male subgroup: 95 matched pairs per group — designated as exploratory due to suboptimal balance for BMI (SMD 0.273) and smaller sample size. Values are Mean ± SD or n (%).

a Events below TriNetX privacy reporting threshold (<11 per group). NAFLD, non-alcoholic fatty liver disease; GERD, gastro-esophageal reflux disease.

## 4 Discussion

This propensity score-matched analysis of 636 patients from the TriNetX US Collaborative Network demonstrates that adolescents and young adults with severe obesity achieve equivalent 1-year outcomes following laparoscopic sleeve gastrectomy across all measured domains, including primary weight loss, persistence of metabolic comorbidities, new-onset GERD, and early surgical safety. These findings provide population-level evidence that age at SG, within the 13–21-year transition window, does not independently determine short-term bariatric outcomes when patient characteristics — including, crucially, pre-surgical GLP-1 RA exposure — are rigorously balanced.

Our primary finding of equivalent BMI reduction (∼18–19% from baseline at 1 year) aligns closely with the existing adolescent bariatric literature and, most directly, with the only comparable age-stratified analyses published to date. In the Teen-LABS consortium sub-analysis by Ogle and colleagues, younger (13–15 years) and older (16–19 years) adolescents undergoing metabolic and bariatric surgery achieved nearly identical 5-year BMI reductions (−22.2% vs −24.6%), with no significant differences in remission of hypertension or dyslipidemia or in quality of life [17]Similarly, the matched case-control Tehran Obesity Treatment Study (TOTS) reported comparable 1-year outcomes between adolescents and young adults following bariatric surgery [18] A single-center 5-year comparison from Qatar likewise found no significant difference in weight loss between adolescents and adults undergoing SG, despite nearly identical baseline BMI (47.6 vs 48.4 kg/m^2^) — figures remarkably concordant with our own matched cohort (47.3 vs 48.4 kg/m^2^).^[7]^ The convergence of our population-level, propensity-matched findings with these prospective and single-center datasets strengthens confidence that age at SG, within the 13–21 year window, does not materially alter short-term weight outcomes. The somewhat smaller absolute reductions in our EHR-based cohort reflect the nature of the most recent recorded measurement within a 1-year window rather than a standardized post-operative visit, and the heterogeneity inherent in real-world practice.

The most methodologically significant contribution of this study is the identification and correction of semaglutide as a major pre-treatment confounder in adolescent bariatric research. We found that 29.4% of adolescents had received pre-surgical semaglutide, compared with only 14.6% of young adults — a nearly 2-fold disparity driven by the 2022 FDA approval of semaglutide for patients aged 12 and older following the STEP TEENS trial [10,11]Pre-surgical GLP-1 RA use is physiologically relevant to bariatric outcomes: these agents independently reduce BMI by 15–18%, improve insulin sensitivity, and reduce hepatic steatosis, each of which directly influences the outcome metrics central to bariatric effectiveness studies [11,12] Failure to control for this exposure in any study comparing adolescent and adult bariatric outcomes risks attributing GLP-1 RA-related pre-operative improvements to age-related biological differences. After PSM incorporating semaglutide, balance was achieved (SMD 0.028), and the resulting outcome equivalence can be interpreted with greater confidence. We urge future comparative studies in adolescent bariatric populations to routinely document and adjust for GLP-1 RA pre-treatment.

The high prevalence of vitamin D insufficiency — affecting approximately 49% of patients in both cohorts prior to surgery — represents a clinically actionable secondary finding that is independent of the primary comparison. Pre-existing hypovitaminosis D in severe obesity is well documented, driven by adipose tissue sequestration of the fat-soluble vitamin and by reduced sun exposure associated with physical inactivity.^[8]^ Notably, a recent large TriNetX analysis of metabolic surgery patients reported strikingly similar post-operative nutritional deficiency rates (vitamin D 52.4%, vitamin B12 18.0%, iron deficiency 11.5%), and confirmed that SG was associated with fewer nutritional complications than RYGB [16] externally corroborating both the magnitude of nutritional risk in our cohort and the validity of our network-based methodology. Post-operative vitamin D deficiency typically worsens after SG due to reduced gastric acid secretion, which impairs D3 absorption, and the deficiency may not appear in EHR records until 2–4 years post-operatively [16]Our data underscore the importance of universal pre-operative vitamin D assessment and aggressive supplementation in all adolescent and young adult bariatric surgery candidates, consistent with current ASMBS nutritional guidelines [19,20].

The null finding for new-onset GERD (6.2% vs 6.4%) is clinically meaningful in the context of ongoing debate about SG and reflux. Critics of SG in adolescents have raised concern that the procedure worsens or induces GERD — a complication with potentially greater lifetime impact in younger patients given the decades of exposure ahead [21].Our finding that new GERD incidence at 1 year is not higher in adolescents compared with young adults is reassuring, though it does not preclude longer-term divergence; GERD progression after SG may not manifest until 3–5 years post-operatively and warrants prospective monitoring in adolescent cohorts.

The metabolic comorbidity persistence data — T2D in 10.1% vs 11.0%, hypertension in 9.1% vs 11.0% suggest a consistent pattern of slightly favorable (though non-significant) outcomes in adolescents for metabolic endpoints. This is biologically plausible: younger patients with a shorter duration of metabolic disease and preserved beta-cell reserve may achieve better postoperative glycemic remission, consistent with the known age-dependent reversibility of T2D in bariatric population [17,22] Of note, the Ogle Teen-LABS sub-analysis observed the inverse direction for diabetes specifically — younger adolescents were somewhat less likely to achieve T2D remission — while simultaneously demonstrating a trend toward fewer nutritional deficiencies in younger patients, possibly reflecting greater parental supervision of supplementation within the home environment [17] These divergent age-related signals for diabetes across datasets underscore that T2D-specific responses may be sensitive to cohort composition and follow-up duration. With n = 318 per group and an event rate of approximately 10%, our study was powered to detect differences of approximately 6 percentage points with 80% power; smaller true differences may exist but remain undetectable at this sample size.

## 5 Limitations

This study has several limitations that should be considered when interpreting the findings. First, the median follow-up for adolescents (301 days) was shorter than for young adults (365 days), an asymmetry inherent to the younger cohort's more recent surgical dates. While we pre-specified the 365-day window to minimize this disparity, residual differential censoring may influence Kaplan-Meier estimates. Second, ferritin (SMD 0.363) and triglycerides (SMD 0.237) showed residual imbalance after matching, as these were observational rather than PSM covariates; nutritional outcome comparisons may be partially confounded. Third, TriNetX captures EHR-documented events and does not capture procedures performed at non-participating facilities, out-of-network follow-up visits, or patient-reported outcomes, potentially underestimating reoperation and complication rates. Fourth, ICD-10 and CPT coding accuracy depends on institutional coding practices and may introduce misclassification. Fifth, one or more healthcare organizations failed to return results in all analyses, introducing potential geographic or institutional bias of unknown direction. Sixth, the male subgroup analysis (n = 95 matched pairs) was limited by suboptimal BMI balance (SMD 0.273) and should be interpreted as exploratory. Seventh, the 1-year observation window precludes conclusions about long-term weight regain, late nutritional deficiencies (iron, vitamin D, protein), reoperation rates, and quality-of-life outcomes — domains that are critical for fully characterizing the impact of age at SG and that require prospective, registry-based studies with minimum 3–5 year follow-up.

## 6 Conclusion

Laparoscopic sleeve gastrectomy produces equivalent 1-year weight loss and metabolic outcomes in adolescents (13–17 years) and young adults (18–21 years) with severe obesity, even after rigorous propensity score adjustment for the substantially higher rate of pre-surgical GLP-1 receptor agonist use in younger patients. These findings support the clinical safety and efficacy of SG across this age transition window and highlight pre-surgical semaglutide use as an important, previously uncontrolled confounder in comparative bariatric research involving adolescents. The near-universal presence of vitamin D insufficiency in both age groups underscores the need for systematic pre-operative micronutrient screening and supplementation. Prospective, registry-based studies with extended follow-up are essential to characterize the long-term nutritional, reoperation, and quality-of-life outcomes of SG in adolescents.

## Key takeaways

● Adolescents and young adults achieve equivalent 1-year weight loss and metabolic remission after sleeve gastrectomy, supporting the procedure's efficacy across the 13–21 year age transition window.
● Pre surgical GLP-1 receptor agonist use, particularly semaglutide, is significantly more common among adolescents and must be adjusted for as a confounder in all comparative bariatric outcomes research
● Vitamin D insufficiency affect approximately half of adolescents and young adults bariatric candidates per-operatively necessitating routine screening and aggressive supplementation prior to surgery.

## Ethics statement

This study utilized de-identified, aggregated data from the TriNetX Research Network. In accordance with federal regulations (45 CFR 46.101(b) [4]), institutional review board approval was not required.

## Data availability

Aggregate-level data were generated through the TriNetX analytics platform and are available upon reasonable request to the corresponding author, subject to TriNetX data use agreement provisions.

## Author contributions

**Ahmed Taki-Eldin**: Conceptualization; Methodology; Formal analysis; Writing – original draft; Visualization; Project administration. **Ahmed Lotfy**: Conceptualization; Methodology; Validation; Writing – review & editing; Supervision; Formal analysis. **Mohamed saif Eldin Mahmoud El-Soudani**: Methodology; Investigation; Data curation; Writing – original draft; Writing – review & editing; Validation. **Mahmoud Ghaly**: Methodology; Investigation; Data curation; Writing – original draft; Writing – review & editing; Visualization. **Mohamed Mahmoud Mohamed Eltaweel**: Investigation; Data curation; Validation; Writing – review & editing. **Eslam Taha Ghalwash**: Investigation; Data curation; Validation; Writing – review & editing. **Tamer Rushdy Elalfy**: Investigation; Data curation; Formal analysis; Writing – review & editing; Software. **Ahmed M.Kandel**: Investigation; Data curation; Writing – review & editing; Software. **Zakaria M Abdel Sadek**: Investigation; Data curation; Writing – review & editing; Supervision. **Ahmed Naji Ahmed**: Data curation; Writing – review & editing; Visualization.

## Artificial intelligence declaration

We declare that no artificial intelligence (AI) or generative AI tools were used in the writing of this research paper.

## Funding

This study received no external funding. The TriNetX platform was accessed through institutional licensing.

## Conflict of interest disclosure

The authors declare no conflicts of interest.

## References

[1] Fryar Cheryl D.C., Margaret D., Afful Joseph (2020). https://www.cdc.gov/nchs/data/hestat/obesity-child-17-18/obesity-child.htm.

[2] Pratt J.S.A., Browne A., Browne N.T., Bruzoni M., Cohen M., Desai A. (2018 Jul). ASMBS pediatric metabolic and bariatric surgery guidelines, 2018. Surg Obes Relat Dis.

[3] Griggs C.L., Perez N.P., Goldstone R.N., Kelleher C.M., Chang D.C., Stanford F.C. (2018 Dec 1). National trends in the use of metabolic and bariatric surgery among pediatric patients with severe obesity. JAMA Pediatr.

[4] Inge T.H., Courcoulas A.P., Jenkins T.M., Michalsky M.P., Helmrath M.A., Brandt M.L. (2016 Jan 14). Weight loss and health status 3 years after bariatric surgery in adolescents. N Engl J Med.

[5] Inge T.H., Jenkins T.M., Xanthakos S.A., Dixon J.B., Daniels S.R., Zeller M.H. (2017 Mar). Long-term outcomes of bariatric surgery in adolescents with severe obesity (FABS-5+): a prospective follow-up analysis. Lancet Diabetes Endocrinol.

[6] Olbers T., Beamish A.J., Gronowitz E., Flodmark C.E., Dahlgren J., Bruze G. (2017 Mar). Laparoscopic Roux-en-Y gastric bypass in adolescents with severe obesity (AMOS): a prospective, 5-year, Swedish nationwide study. Lancet Diabetes Endocrinol.

[7] Khidir N., El-Matbouly M.A., Sargsyan D., Al-Kuwari M., Bashah M., Gagner M. (2018 Jul). Five-year outcomes of laparoscopic sleeve gastrectomy: a comparison between adults and adolescents. Obes Surg.

[8] Xanthakos S.A. (2009 Oct). Nutritional deficiencies in obesity and after bariatric surgery. Pediat Clin North Am.

[9] Skinner A.C., Perrin E.M., Moss L.A., Skelton J.A. (2015 Oct). Cardiometabolic risks and severity of obesity in children and young adults. N Engl J Med.

[10] U.S. Food and Drug Administration (2022 Dec). FDA approves new drug treatment for chronic weight management in children aged 12 years and older [internet]. https://www.fda.gov/.

[11] Weghuber D., Barrett T., Barrientos-Pérez M., Gies I., Hesse D., Jeppesen O.K. (2022 Dec 15). Once-weekly semaglutide in adolescents with obesity. N Engl J Med.

[12] Kelly A.S., Auerbach P., Barrientos-Perez M., Gies I., Hale P.M., Marcus C. (2020 May 28). A randomized, controlled trial of liraglutide for adolescents with obesity. N Engl J Med.

[13] Von Elm E., Altman D.G., Egger M., Pocock S.J., Gøtzsche P.C., Vandenbroucke J.P. (2007 Oct). The Strengthening the Reporting of Observational Studies in Epidemiology (STROBE) statement: guidelines for reporting observational studies. Lancet.

[14] Rosenbaum P.R., Rubin D.B. (1983). The central role of the propensity score in observational studies for causal effects. Biometrika.

[15] Austin P.C. (2011 May 31). An introduction to propensity score methods for reducing the effects of confounding in observational studies. Multivariate Behav Res.

[16] Zhu X., Wang Q., Mocanu V., Kachornvitaya P., Wills M.V., Strong A. (2026 Feb). Evaluating the long-term safety and disease-modifying potential of metabolic surgery in patients with rheumatoid arthritis: a TriNetX cohort study. Surg Obes Relat Dis.

[17] Ogle S.B., Dewberry L.C., Jenkins T.M., Inge T.H., Kelsey M., Bruzoni M. (2021 Mar 1). Outcomes of bariatric surgery in older versus younger adolescents. Pediatrics.

[18] Aryannezhad S., Hosseinpanah F., Khalaj A., Mahdavi M., Valizadeh M., Akhavirad S.M.B. (2021 Nov). Comparison of the one-year outcomes of bariatric surgery in adolescents and young adults: a matched case–control study, Tehran Obesity Treatment Study (TOTS). Surg Today.

[19] Mechanick J.I., Apovian C., Brethauer S., Garvey W.T., Joffe A.M., Kim J. (2020 Feb). Clinical practice guidelines for the perioperative nutrition, metabolic, and nonsurgical support of patients undergoing bariatric procedures – 2019 update: cosponsored by American association of clinical endocrinologists/american college of endocrinology, the obesity society, American society for metabolic & bariatric surgery, obesity medicine association, and American society of anesthesiologists. Surg Obes Relat Dis.

[20] Parrott J., Frank L., Rabena R., Craggs-Dino L., Isom K.A., Greiman L. (2017 May). American society for metabolic and bariatric surgery integrated health nutritional guidelines for the surgical weight loss patient 2016 update: micronutrients. Surg Obes Relat Dis.

[21] Stenard F. (2015). Laparoscopic sleeve gastrectomy and gastroesophageal reflux. WJG.

[22] Michalsky M.P., Inge T.H., Jenkins T.M., Xie C., Courcoulas A., Helmrath M. (2018 Feb 1). Cardiovascular risk factors after adolescent bariatric surgery. Pediatrics.

